# Surgical Timing After Acromioclavicular Joint Separation: Association With Clinical Outcomes

**DOI:** 10.1177/23259671261438133

**Published:** 2026-05-05

**Authors:** Mallory B. Lane, Jacob Siahaan, James M. Gregory

**Affiliations:** † Department of Orthopedic Surgery, The University of Texas Health Science Center at Houston, McGovern Medical School, Houston, Texas, USA; Investigation performed at The University of Texas Health Science Center at Houston, McGovern Medical School, Houston, Texas, USA

**Keywords:** acromioclavicular joint, acromioclavicular joint reconstruction, acromioclavicular joint repair, acromioclavicular joint separation

## Abstract

**Background:**

Treatment of acromioclavicular joint (ACJ) separations continually lacks consensus between providers, and while low-grade injuries generally respond well to conservative management, high-grade injury management is more complex. For patients requiring surgery, the effect of the timing of surgery with respect to the original injury on clinical outcomes remains unclear.

**Hypothesis:**

Clinical outcome measures would worsen as the time between ACJ dislocation injury and surgery increased.

**Study Design:**

Cohort study; Level of evidence, 3.

**Methods:**

A large nationwide insurance claims database was queried for patients who had an ACJ dislocation injury using International Classification of Diseases, Ninth and Tenth Revision (ICD-9 and ICD-10) codes. Surgical management via ACJ repair or ACJ reconstruction was identified using Current Procedural Terminology codes 23550 and 23552, respectively. The time between the date of initial ACJ dislocation diagnosis and the date of surgery was used to separate patients into 4 temporal subgroups with the following parameters: 0 to 4 weeks, 4 weeks to 3 months, 3 months to 1 year, and after 1 year. Clinical outcomes—including complication rate, infection rate, and fracture rate—were compared between temporal and procedural subgroups. Outcomes were compared using chi-square tests.

**Results:**

A total of 13,194 patient met the inclusion criteria and were included in the study, of whom 8639 received surgical management of their ACJ dislocation within 4 weeks of initial injury diagnosis, 2210 between 4 weeks and 3 months, 1264 between 3 months and 1 year, and 1081 more than 1 year after injury diagnosis. The rate of all measured adverse clinical outcomes increased over time as surgical management was delayed in the ACJ repair subgroup, and the rates of revisions and complications increased over time as surgical management was delayed in the ACJ reconstruction subgroup.

**Conclusion:**

The rate of all measured adverse clinical outcomes increased over time as surgical management was delayed in the ACJ repair subgroup, and the rate of revisions and complications increased over time as surgical management was delayed in the ACJ reconstruction subgroup. Further research is needed to define the role of injury severity and classification in these outcomes and identify which patients would benefit from early surgical intervention.

Despite being one of the most common injuries of the shoulder girdle, accounting for 4% to 12% of injuries, clinical evidence on a superior acromioclavicular joint (ACJ) separation treatment algorithm remains limited in the literature.^17^ Historically, a universal nonoperative approach was used for all ACJ injuries until a transition was made in the 1990s to an approach that focused more on dislocation type.^22,24^ Early manifestations of this idea favored conservative management for low-grade dislocations (less coracoclavicular [CC] displacement), and surgical management for high-grade dislocations (more CC displacement, disruption of both AC and CC ligamentous structures).^22,24^

There is dissent over the currently used classification systems, with critics questioning their accuracy and generalizability and highlighting their reliance on patient-dependent factors, such as muscle contraction and shoulder position, rather than objective anatomical differences.^1,3^ In terms of treatment, there is discussion over the role of surgery and whether the benefits of nonoperative management—including faster return to work and sports activities—are superior or inferior to the possibility of long-term deformity or scapular dyskinesis if surgical management is delayed considerably.^4,15^

The 6-part Rockwood classification system for ACJ separations was first described by Charles Rockwood in 1984 and remains the most used classification system for ACJ separations in the modern era.^9^ Details of each of the Rockwood classification types can be found in Tables 1 and 2, which are adapted from Gorbaty et al.^11^ Rockwood types I and II injuries are considered stable due to the lack of complete tearing of both the CC and ACJ ligaments, whereas Rockwood types III and VI injuries are considered unstable due to disruption of the entire CC and ACJ complex. This disruption is typically diagnosed by >100% displacement on plain radiograph. Although not explicitly defined in the Rockwood classification system, standard radiographic evaluation for ACJ injuries includes anteroposterior (AP) views, axillary views, and Zanca views of both shoulders.^25^ The Zanca view is a specific type of AP radiograph taken with 10° to 15° of cephalic tilt to better visualize the ACJ by removing the scapular spine from the field of view.^25^ Bilateral imaging allows for direct comparison of the injured and uninjured side to more accurately assess the degree of superior displacement of the clavicle and widening of the AC and CC distances to determine Rockwood classification.^25^ Throughout the present study, low-grade ACJ separations will be defined as Rockwood types I and II, and high-grade ACJ separations will be defined as Rockwood types III and VI.

**Table 1:** Description of Low-Grade Rockwood Classifications for ACJ Separations*^*a*^*

	I	II
AC ligament	Sprained	Complete tear
ACJ	Intact	Disrupted; transverse plane widening
CC ligaments	Intact	Sprained; slight interval widening
Deltoid and trapezius muscles	Intact	Possible partial detachment

a ACJ, acromioclavicular joint; CC, coracoclavicular.

**Table 2:** Description of High-Grade Rockwood Classifications for ACJ Separations*^*a*^*

	III	IV	V	VI
AC ligament	Complete tear	Complete disruption	Complete disruption	Complete disruption
ACJ	Dislocated; clavicle displaced superiorly relative to acromion	Dislocated; clavicle displaced posteriorly into or through trapezius	Dislocated; extreme vertical incongruity between the lateral clavicle and the acromion	Dislocated; clavicle displaced inferior relative to acromion
CC ligaments	Disrupted; interval widening ≤ 100%	Partial or complete disruption with a change in interval orientation	Complete disruption; interval widening 100% to 300%	Intact; interval decreased or reversed
Deltoid and trapezius muscles	High probability of detachment from the distal clavicle	High probability of detachment from the distal clavicle	High probability of detachment from the distal clavicle	Intact, partial, or complete detachment

a ACJ, acromioclavicular joint; CC, coracoclavicular.

Although it is now widely accepted that low-grade, stable AC separations can be treated nonoperatively, the role of surgical management of high-grade ACJ separations remains controversial. Recent studies have shown comparable outcomes for nonsurgical management of high-grade injuries, with these patients usually returning to work and sports activities more quickly than those who underwent surgical fixation.^4,15^ Additionally, surgical treatment has been shown to achieve functional outcomes equivalent to nonoperative treatment in Rockwood grade III AC separations, albeit with a slower recovery and higher complication and revision rates.^26^ However, some patients remain symptomatically unstable with nonoperative treatment of high-grade ACJ injuries, and concerns of possible long-term deformity or scapular dyskinesis remain.^4,15^ A proposed algorithm for treatment of these injuries suggests initial nonoperative treatment, followed by reevaluation for surgical candidacy at 6 weeks from injury.^13^ Other studies have noted that patients who underwent surgery acutely (within 21 days of injury) had better patient-reported outcome measures (PROMs) than those whose surgical management was delayed >21 days out from injury.^7^

Even though several studies have attempted to remedy the lack of consensus on the method and timing of treatment for ACJ injuries, these studies have limited generalizability due to small sample sizes and limited scope. Therefore, it remains unclear whether the timing of surgical management for ACJ injuries translates to improved outcomes. Does nonoperative treatment potentially compromise eventual results? Soft tissue healing potential may differ depending on the time from the initial injury. We hypothesized that clinical outcome measures would worsen as the time between ACJ dislocation injury and surgery increased.

## Methods

In this study, datasets were analyzed using the PearlDiver Mariner Patient Claims Database (PearlDiver Technologies). The PearlDiver Mariner 165 database is an all-payer claims database that includes 75.7 billion claims across 165.7 million patients. Payer types include commercial health plans, government plans (including Medicare and Medicaid), employer-sponsored plans, workers' compensation, and patient cash payments. The PearlDiver database includes a vast set of Health Insurance Portability and Accountability Act-compliant patient information that can be queried through the use of current procedure terminology (CPT), International Classification of Diseases (ICD-9 and ICD-10), as well as many other classifications, such as date of treatment/procedure, length of treatment, prescribed medication, et cetera.

Within the PearlDiver database, an initial query was conducted to identify patients with an ACJ separation injury using ICD-9 code 83104 and ICD-10 codes in Supplemental Table 1 (n = 273,971). A query was then completed to identify patients who also had a corresponding ACJ surgery, either an ACJ repair using CPT 23550 (n = 10,526) or an ACJ reconstruction surgery using CPT code 23552 (n = 7067). These initial cohorts were then split into groups based on the time interval between their injury, marked by the date the ACJ separation surgery ICD-9 and ICD-10 codes were first used, and their ACJ surgery, marked by the date the respective CPT code was first used.

The primary outcome of each cohort was revision rate. Given that there is no CPT code for revision ACJ surgery, we defined revision as the CPT code opposite to the patient's initial surgery type. The CPT code for ACJ reconstruction was used to classify revisions for the cohort that initially had an ACJ repair surgery, and the CPT code for ACJ repair was used to classify revisions for the cohort that initially had an ACJ reconstruction surgery. The secondary outcomes of each cohort were complication rate, infection rate, and fracture rate; definitions of which can be found in Supplemental Table 2. Fracture rate was analyzed by overall fracture rate as well as by subgroups for clavicle fractures and coracoid fractures. Outcomes were compared across the 4 temporal cohorts overall and for both the ACJ repair and ACJ reconstruction subgroups, referring to the type of surgery done initially for those who had a revision procedure.

Continuous variables were calculated and reported as the mean ± standard error of the mean. Means were compared with the Welch *t* test. Categorical variables were expressed as frequencies and percentages. Chi-square tests were utilized to compare categorical variables between patient groups. All analyses were performed at an alpha level of .05, indicating statistical significance (*P* < .05).

## Results

A total of 273,971 patients in the PearlDiver database had an ACJ separation injury from January 2015 through March 2022. A total of 13,194 patients met the inclusion criteria and were included in the study. Table 3 shows the breakdown of the 4 temporal cohorts used during analysis as well as the number of patients within each cohort who received ACJ repair as their surgical management (CPT 23550), the number who received ACJ reconstruction for their surgical management (CPT 23552), as well as the overall cohort size, comprised of patients who received either of the 2 previously mentioned surgical procedures in the treatment of their ACJ separation injury.

**Table 3:** Cohort Size for Temporal Groups: Repair Versus Reconstruction

Time From Injury to Surgery	n	Type of Initial Surgery	n
0-4 wk	8639	Repair	5711
		Reconstruction	2928
4 wk-3 mo	2210	Repair	1163
		Reconstruction	1047
3 mo-1 yr	1264	Repair	616
		Reconstruction	648
1 yr +	1081	Repair	527
		Reconstruction	554

### Revision Rate

**Figure 1.**
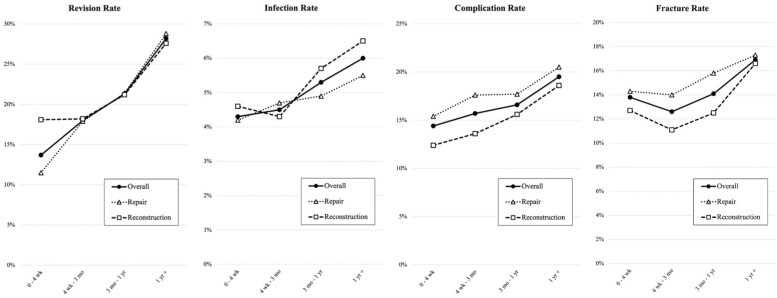
Temporal trends in postoperative outcomes after repair versus reconstruction procedures for ACJ separation injuries. ACJ, acromioclavicular joint.

Significant increases in the overall revision rate were observed at each time point from 0 to 4 weeks, as shown in Table 4 and Figure 1. The rate increased from 13.7% in patients who underwent surgery within 4 weeks of injury to 28.2% in patients who underwent surgery >1 year after injury (*P* < .001). Notably for the overall cohort, the increase in the revision rate over time was statistically significant across all comparison groups (all, *P* < .001 except 1; *P* = .02). Patients who underwent ACJ repair also showed significantly higher revision rates at all time points compared with surgical management in the acute 0 to 4 week time period (11.5% [0-4 wks], 17.9% [4 wk-3 mo], 21.4% [3 mo-1yr], 28.8% [1yr+]; *P* < .001 for all comparisons). In contrast, patients who underwent ACJ reconstruction alone had a significantly higher revision rate when surgery was performed >1 year after injury (18.1% vs 27.6%; *P* < .001).

**Table 4:** Postoperative Outcomes and *P* Values of Compared Temporal Cohorts for ACJ Separation Injuries*^*a*^*

						*P*					
		Raw Data				Control Group			Control Group		Control Group
						0-4 wk			4 wk-3 mo		3 mo-1 yr
						Comparison Group			Comparison Group		Comparison Group
		0-4 wk	4 wk-3 mo	3 mo-1 yr	1 yr +	4 wk-3 mo	3 mo-1 yr	1 yr +	3 mo-1 yr	1 yr +	1 yr +
Revisions	Overall	1186 (13.7)	398 (18)	269 (21.3)	305 (28.2	**<.001** ^*b*^	**<.001** ^*b*^	**<.001** ^*b*^	**.02** ^*d*^	**<.001** ^*b*^	<.001*^b^
	Repair	657 (11.5)	208 (17.9)	132 (21.4)	152 (28.8)	**<.001** ^*b*^	**<.001** ^*b*^	**<.001** ^*b*^	.07^*e*^	**<.001** ^*b*^	.004*^c^
	Reconstruction	529 (18.1)	190 (18.2)	137 (21.2)	153 (27.6)	.95^*e*^	.69^*e*^	**<.001** ^*b*^	.13^*e*^	**<.001** ^*b*^	.009*^c^
Infections	Overall	372 (4.3)	100 (4.5)	67 (5.3)	65 (6)	.66^*e*^	**<.001** ^*b*^	**<.001** ^*b*^	.30^*e*^	.09^*e*^	<.001*^b^
	Repair	237 (4.2)	55 (4.7)	30 (4.9)	29 (5.5)	.42^*e*^	.46^*e*^	**<.001** ^*b*^	.99^*e*^	.50^*e*^	.85^*e*^
	Reconstruction	135 (4.6)	45 (4.3)	37 (5.7)	36 (6.5)	.74^*e*^	.28^*e*^	**.002** ^*d*^	.23^*e*^	.17^*e*^	.85^*e*^
Complications	Overall	1245 (14.4)	347 (15.7)	210 (16.6)	211 (19.5)	.12^*e*^	**<.001** ^*b*^	**<.001** ^*b*^	.48^*e*^	**.002** ^*c*^	.06^*e*^
	Repair	881 (15.4)	205 (17.6)	109 (17.7)	108 (20.5)	.07^*e*^	.16^*e*^	**<.001** ^*b*^	**≥.9999** ^*e*^	**.04** ^*d*^	.88^*e*^
	Reconstruction	364 (12.4)	142 (13.6)	101 (15.6)	103 (18.6)	.37^*e*^	**.04** ^*d*^	**<.001** ^*b*^	.28^*e*^	**.02** ^*d*^	.38^*e*^
Fractures	Overall	1188 (13.8)	279 (12.6)	178 (14.1)	183 (16.9)	.17^*e*^	**<.001** ^*b*^	**<.001** ^*b*^	.22^*e*^	**.003** ^*c*^	.06^*e*^
	Repair	817 (14.3)	163 (14)	97 (15.8)	91 (17.3)	.57^*e*^	.64^*e*^	**<.001** ^*b*^	.44^*e*^	.07^*e*^	.89^*e*^
	Reconstruction	371 (12.7)	116 (11.1)	81 (12.5)	92 (16.6)	.23^*e*^	.73^*e*^	**<.001** ^*b*^	.64^*e*^	**.01** ^*c*^	.15^*e*^

a Data are presented as n (%), unless otherwise specified. Bold *P* values indicate statistical significance. ACJ, acromioclavicular joint.

b *P* < .001.

c .001 < *P*≤ .01.

d .01 < *P*≤ .05.

e Not significant.

### Secondary Outcomes

Secondary outcomes (infection, complications, and fractures) worsened as time to surgery increased in the overall cohort and within repair and reconstruction subgroups (Figure 1). Overall infection rate increased significantly from 4.3% in acutely treated injuries (0-4 weeks) to 6% in patients treated ≥1 year after injury (*P* < .001). This finding persisted whether patients underwent repair or reconstruction (repair: 4.2% [0-4wks] to 5.5% [1yr +]; reconstruction: 4.6% [0-4wks] to 6.5% [1yr+]; *P* < .002 in both). Overall complication rate significantly increased from 14.4% in acutely treated injuries (0-4 weeks) to 19.5% in patients treated ≥1 year after injury (*P* < .001). This finding persisted whether patients underwent repair or reconstruction (repair: 15.4% [0-4wks] to 20.5% [1yr +]; reconstruction: 12.4% [0-4wks] to 18.6% [1yr+]; *P* < .001 in both). Overall fracture rate significantly increased from 13.8% in acutely treated injuries (0-4 weeks) to 16.9% in patients treated over a year from injury (*P* < .001). This finding persisted whether patients underwent repair or reconstruction (repair: 14.3% [0-4wks] to 17.3% [1yr +]; reconstruction: 12.7% [0-4wks] to 16.6% [1yr+]; *P* < .001 in both).

## Discussion

In our study, patients who underwent surgical management of their ACJ separation within 4 weeks of injury, subsequently referred to as acute surgical management, were found to have improved patient outcomes—including lower rates of complications, infections, fractures, and need for revision—when compared with patients who underwent surgery over 1 year after their injury, subsequently referred to as chronic surgical management. In general, adverse outcomes increased with increasing interval between injury and surgery among patients who underwent ACJ repair, except for fracture rate in the 0-4 wk cohort versus the 4 wk-3 mo cohort (14.3% vs 14%). For the ACJ repair subgroup, there is a statistically significant improvement in all measured patient outcomes in the acute surgical management cohort compared with both the 3 mo-1yr and 1yr+ cohorts. In the ACJ reconstruction subgroup, revision rate increased at each time point; however, it was statistically significant only when comparing the acute surgical management cohort with the chronic surgical management cohort. The complication rate also increased at each time point for the ACJ reconstruction subgroup and was statistically significant when comparing the 0-4 wk cohort with both the 3 mo-1yr and 1yr+ cohorts. It is, however, difficult to determine the clinical significance of this finding as the other major complications included in this analysis, that is, infection and fracture rate, were not significant at time points <1 year. In summary, ACJ repair is viable only in the acute phase after injury, while ACJ reconstruction remains viable until 1 year after injury. As tissue healing potential decreases after the acute phase of injury, these findings support the consensus that ACJ reconstruction may be better suited for surgical management of chronic ACJ injuries. Overall, our findings confirm our hypothesis that a longer time interval between ACJ dislocation injury and surgical management negatively impacts clinical outcomes.

Previous studies examining various clinical outcome measures for ACJ repair and reconstruction surgery have shown comparable complication rates but substantially lower revision rates. The sample sizes of these studies, however, have also been appreciably smaller than that of the present study. A 2018 study reviewed complication rates and unplanned reoperation rates for various surgical treatment methods for ACJ instability—including free graft reconstruction, suspensory devices, synthetic ligament devices, modified Weaver-Dunn, and hook plate/K-wires techniques with a study sample size of n = 939 patients.^19^ Complication rates were 10.3%, 6.2%, 4.4%, 12.8%, and 26.3%, and unplanned reoperation rates were 1.2%, 2.8%, 0.9%, 5.4%, and 2.6% in free tendon graft, suspensory devices, synthetic ligament devices, modified Weaver-Dunn, and hook plate/K-wires techniques, respectively.^19^ Another 2018 study reviewed an 11-year period using the American Board of Orthopaedic Surgery database and found an overall complication rate of 24.5% and a reoperation rate of 7.3% across various surgical management options used for ACJ separations, based on 1447 patients who met the inclusion criteria.^14^ Another study^12^ that reviewed 1704 patients undergoing ACJ reconstruction surgery found an overall complication rate of 14.2%, with infection, fracture, and revision rates of 6.3%, 5.7%, and 9.5%, respectively. Of previous studies on the subject, the one with the largest sample size yielded results most similar to those of the present study. Wang et al^28^ (n = 2106) found a revision rate of 10.9% for ACJ reconstruction within 6 months of initial surgery.^28^ Several previous studies have also suggested that a longer time interval between injury and surgical management may have negative consequences for patient outcomes. Chen et al^6^ found that patients who had surgery >6 weeks after injury had a significantly greater risk of complication (odds ratio [OR], 3.19 [95% CI, 1.34-7.77]; *P* = .009) as well as a significantly greater risk of structural failure (OR, 2.65 [95% CI, 1.38-5.28]; *P* = .004). Similarly, a 2023 study noted that patients who underwent surgery acutely (within 21 days of injury) had better PROMs than those whose surgical management was delayed >21 days from injury.^7^ A 2024 study determined that the timing of ACJ surgery did not affect functional outcomes in patients with high-grade ACJ separation, defined as Rockwood grade III and V.^23^ Outcome measures included complications, revisions, American Shoulder and Elbow Surgeons score, Single Assessment Numeric Evaluation scores, and radiographic outcomes, which were used to measure the CC distance at various stages.^23^ This study also used 4 weeks after injury as the determining factor for early versus delayed surgical intervention, similarly to the present study. However, because only 221 patients were included, the study was not powered to detect differences in loss of reduction or complication rates, as the present study was.^23^

An important aspect of ACJ separation management is deciding whether to perform an ACJ repair or reconstruction, and determining the optimal timing for surgical intervention. As evidenced by ongoing expert debate, this decision is complex and nuanced. Several key factors to consider for each patient include age, comorbidities, functional demand versus current functionality, and hand dominance, among many others. Knowledge of the ligamentous healing potential of the acromioclavicular ligament complex (ACLC) remains rather limited.^5,10,18,20,21,27^ A 2020 study examined the healing potential of the ACLC in patients who underwent surgical management with ACJ reconstruction within 21 days of ACJ injury; they found that the ACLC showed ligamentous healing at a mean of 12 weeks after surgery.^27^ This study, however, only looked at ligamentous healing potential for acute injuries undergoing surgical management within 3 weeks of injury. ^27^ Maier et al^16^ found that the AC ligament exhibited early and dynamic healing responses after traumatic rupture, with histological findings suggesting that the healing potential of the AC ligament is highest within the first week after injury; therefore, this timeframe is most favorable for surgical intervention. The study went on to define the phases of ligamentous healing, finding that the first week after injury is the acute inflammatory phase, characterized by an increase in total cell count and CD68-positive cells as well as metabolic activation of fibrocyte-like cells, which make up the physiology behind the increased healing potential of the AC ligament.^16^ It is also important to note that what constitutes the “acute” timeframe after injury varies throughout the literature.^8^ A 2014 study on common sports injuries found that orthopaedic surgeons in the United States most commonly defined the acute timeframe as <3 weeks from injury and the chronic timeframe as >6 weeks for ACJ dislocations.^7^ While most common, these definitions are far from universally accepted and used. In the present study, acute surgical management of ACJ separation is defined as <4 weeks (<28 days) from injury. A 2025 study^2^ used the Delphi method to gather expert opinions from 18 fellowship-trained surgeons on various topics of ACJ injury management. Consensus was reached on the treatment of subacute and chronic ACJ instability with tendon augmentation with allograft; however, agreement was not reached on the use of autograft tissue. Our study revealed that injury management with either surgical technique within 4 weeks of injury was associated with improved clinical outcome measures compared with delayed surgical intervention. However, the degree of delay in which these improved measures were significant varied between the repair and reconstruction cohorts. When considering the 0-4 wk group in the repair cohort, the rate of revision was significantly less compared with all 3 subsequent time intervals. These results could be explained by the concept that the healing response worsens over time.

As with all database studies that rely on diagnosis and procedure codes rather than individual chart review, our work was limited by the accuracy of recorded data. As a result, we cannot directly confirm the patient's date of injury and must use the date of first diagnosis as a proxy. Inaccurate coding of surgical procedures could alter subgroup analysis and the determination of revision rate. Considering the superior postoperative outcomes of those who underwent surgical management acutely after injury, it is possible that these ACJ separations were less severe and may have been successfully treated with nonoperative treatment. Perhaps only the most severe injuries lead to persistent symptoms >1 year after injury, and our data reflects selection bias. We do not have information about the severity of the ACJ injury in each patient thus, within the confines of a database study, we cannot substantiate this claim. The inability to determine the severity of each patient's injury means it remains unknown whether the injury severities in the repair and reconstruction cohorts were comparable. Similarly, the constraints of our study design limit our ability to ascertain the rationale each surgeon used in choosing the surgical method in various cases, as well as which method was ultimately used. It is important to note that ACJ reconstruction encompasses multiple surgical approaches, some of which require bone tunneling, while others are tunnel-free. This distinction is clinically relevant, as tunnel-free approaches are likely to be associated with lower fracture rates than techniques that require bone tunneling. The present study, however, was unable to ascertain which surgical technique was used for each patient and therefore could not determine the subsequent effect on fracture rate. This study is subject to limitations inherent to administrative coding. In particular, reconstruction procedures could not be reliably stratified by graft type, as available CPT codes do not distinguish between autograft and allograft utilization. As such, no assumption regarding graft source was made in the analysis. This limitation should be considered when interpreting infection risk, as graft type may independently influence postoperative infection rates. Another limitation of the present study is the inability to quantify the number of infections that required further surgery with incision and drainage, as we could not confirm whether they were directly linked to the previous ACJ surgery. Similarly, there may be failures that do not undergo revision surgery, as this study could not quantify failure as other studies have (ie, percentage of displacement compared to the initial postoperative visit). Despite these limitations, this study provides valuable information regarding the surgical management of ACJ injuries. These injuries are relatively uncommon, and the literature remains sparse relative to other shoulder pathologies. As a result, the power of large-scale database analysis is ideal for examining outcomes such as complications and revisions. Looking at national cohorts, with an inherent mix of hospitals and surgeons across a wide geographic area and diverse demographics, allows for generalizable results that can both stimulate further research and affect clinical practice. Database analysis is also time-effective, enabling temporal analysis and long-term follow-up with the availability of longitudinal data.

Based on our study, it appears that “watchful waiting” may have potential negative consequences in the treatment of ACJ separations, especially when ACJ repair is being considered for surgical management rather than ACJ reconstruction. The rate of all measured clinical outcomes increased over time as surgical management was delayed in the ACJ repair subgroup, and the rate of revisions and complications increased over time as surgical management was delayed in the ACJ reconstruction subgroup. Instead of a standardized period of “watchful waiting” for all ACJ injuries, we believe that further research is necessary to better identify which patients would benefit from nonoperative treatment, and which may benefit from a more acute surgical approach. Better understanding and classification of ACJ injuries is likely necessary to predict which patients are anticipated to remain unstable despite nonoperative treatment, and multicenter prospective studies will likely be the best way to provide clinical guidance.

## Conclusion

Patients who received surgical intervention of their ACJ dislocation over a year after initial injury had an increased revision, infection, complication, and fracture rate compared with patients who received surgical management within 4 weeks of injury. We believe these findings highlight the importance of determining which patients would benefit from timely surgical intervention and suggest that observation may not be the preferred first-line treatment for all high-grade ACJ injuries.

## Appendix

**Supplemental Table 1:** ICD-10 Codes Used to Identify Patients with an ACJ Separation Injury

ICD-10 Code	Description
S43.101A	Unspecified dislocation of right acromioclavicular joint, initial encounter
S43.102A	Unspecified dislocation of left acromioclavicular joint, initial encounter
S43.109A	Unspecified dislocation of unspecified acromioclavicular joint, initial encounter
S43.111A	Subluxation of right acromioclavicular joint, initial encounter
S43.112A	Subluxation of left acromioclavicular joint, initial encounter
S43.119A	Subluxation of unspecified acromioclavicular joint, initial encounter
S43.121A	Dislocation of right acromioclavicular joint, 100%-200% displacement, initial encounter
S43.122A	Dislocation of left acromioclavicular joint, 100%-200% displacement, initial encounter
S43.129A	Dislocation of unspecified acromioclavicular joint, 100%-200% displacement, initial encounter
S43.131A	Dislocation of right acromioclavicular joint, greater than 200% displacement, initial encounter
S43.132A	Dislocation of left acromioclavicular joint, greater than 200% displacement, initial encounter
S43.139A	Dislocation of unspecified acromioclavicular joint, greater than 200% displacement, initial encounter
S43.141A	Inferior dislocation of right acromioclavicular joint, initial encounter
S43.142A	Inferior dislocation of left acromioclavicular joint, initial encounter
S43.149A	Inferior dislocation of unspecified acromioclavicular joint, initial encounter
S43.151A	Posterior dislocation of right acromioclavicular joint, initial encounter
S43.152A	Posterior dislocation of left acromioclavicular joint, initial encounter
S43.159A	Posterior dislocation of unspecified acromioclavicular joint, initial encounter

**Supplemental Table 2:** Procedure and Diagnosis Codes Used to Identify Adverse Clinical Outcome Measures

Revisions			
CPT		23550	Open treatment of acromioclavicular dislocation, acute or chronic
		23552	Open treatment of acromioclavicular dislocation, acute or chronic; with fascial graft
Infections			
ICD-9		998.51	Infected postoperative seroma
		998.59	Other postoperative infection
ICD-10		T81.4	Infection following a procedure
		T84.5	Infection and inflammatory reaction due to internal joint prosthesis
		T84.6	Infection and inflammatory reaction due to internal fixation device
		T84.7	Infection and inflammatory reaction due to other internal orthopedic prosthetic devices, implants and grafts
Complications			
ICD-9		996.40	Unspecified mechanical complication of internal orthopedic device, implant, and graft
		996.41	Mechanical loosening of prosthetic joint
		996.42	Dislocation of prosthetic joint
		996.43	Broken prosthetic joint implant
		996.44	Peri-prosthetic fracture around prosthetic joint
		996.45	Peri-prosthetic osteolysis
		996.46	Articular bearing surface wear of prosthetic joint
		996.47	Other mechanical complication of prosthetic joint implant
		996.49	Other mechanical complication of other internal orthopedic device, implant, and graft
		996.77	Other complications due to internal joint prosthesis
		996.78	Other complications due to other internal orthopedic device, implant, and graft
		996.79	Other complications due to other internal prosthetic device, implant, and graft
		998.11	Hemorrhage complicating a procedure
		998.12	Hematoma complicating a procedure
		998.13	Seroma complicating a procedure
		998.30	Disruption of wound, unspecified
		998.31	Disruption of internal operation (surgical) wound
		998.32	Disruption of external operation (surgical) wound
		998.4	Foreign body accidentally left during a procedure
		998.6	Persistent postoperative fistula
		998.89	Other specified complications of procedures not elsewhere classified
ICD-10		T81.1	Postprocedural shock
		T81.3	Disruption of wound, not elsewhere classified
ICD-10 cont.		T81.5	Complications of foreign body accidentally left in body following procedure
		T81.6	Acute reaction to foreign substance accidentally left during a procedure
		T81.7	Vascular complications following a procedure, not elsewhere classified
		T81.8	Other complications of procedures, not elsewhere classified
		T81.9	Unspecified complication of procedure
		T84.0	Mechanical complication of internal joint prosthesis
		T84.1	Mechanical complication of internal fixation device of bones of limb
		T84.2	Mechanical complication of internal fixation device of other bones
		T84.3	Mechanical complication of other bone devices, implants and grafts
		T84.4	Mechanical complication of other internal orthopedic devices, implants and grafts
		T84.8	Other specified complications of internal orthopedic prosthetic devices, implants and grafts
		T84.9	Unspecified complication of internal orthopedic prosthetic device, implant
Fractures			
Clavicle Fracture	ICD-9	810.00	Closed fracture of clavicle, unspecified part
		810.01	Closed fracture of sternal end of clavicle
		810.02	Closed fracture of shaft of clavicle
		810.03	Closed fracture of acromial end of clavicle
	ICD-10	S42.001A	Fracture of unspecified part of right clavicle, initial encounter for closed fracture
		S42.001D	Fracture of unspecified part of right clavicle, subsequent encounter for fracture with routine healing
		S42.001G	Fracture of unspecified part of right clavicle, subsequent encounter for fracture with delayed healing
		S42.001K	Fracture of unspecified part of right clavicle, subsequent encounter for fracture with nonunion
		S42.001P	Fracture of unspecified part of right clavicle, subsequent encounter for fracture with malunion
		S42.001S	Fracture of unspecified part of right clavicle, sequela
		S42.002A	Fracture of unspecified part of left clavicle, initial encounter for closed fracture
		S42.002D	Fracture of unspecified part of left clavicle, subsequent encounter for fracture with routine healing
		S42.002G	Fracture of unspecified part of left clavicle, subsequent encounter for fracture with delayed healing
		S42.002K	Fracture of unspecified part of left clavicle, subsequent encounter for fracture with nonunion
		S42.002P	Fracture of unspecified part of left clavicle, subsequent encounter for fracture with malunion
		S42.002S	Fracture of unspecified part of left clavicle, sequela
		S42.009A	Fracture of unspecified part of unspecified clavicle, initial encounter for closed fracture
		S42.009D	Fracture of unspecified part of unspecified clavicle, subsequent encounter for fracture with routine healing
		S42.009G	Fracture of unspecified part of unspecified clavicle, subsequent encounter for fracture with delayed healing
		S42.009K	Fracture of unspecified part of unspecified clavicle, subsequent encounter for fracture with nonunion
		S42.009P	Fracture of unspecified part of unspecified clavicle, subsequent encounter for fracture with malunion
Clavicle Fracture cont.	ICD-10 cont.	S42.009S	Fracture of unspecified part of unspecified clavicle, sequela
		S42.011A	Anterior displaced fracture of sternal end of right clavicle, initial encounter for closed fracture
		S42.011D	Anterior displaced fracture of sternal end of right clavicle, subsequent encounter for fracture with routine healing
		S42.011G	Anterior displaced fracture of sternal end of right clavicle, subsequent encounter for fracture with delayed healing
		S42.011K	Anterior displaced fracture of sternal end of right clavicle, subsequent encounter for fracture with nonunion
		S42.011P	Anterior displaced fracture of sternal end of right clavicle, subsequent encounter for fracture with malunion
		S42.011S	Anterior displaced fracture of sternal end of right clavicle, sequela
		S42.012A	Anterior displaced fracture of sternal end of left clavicle, initial encounter for closed fracture
		S42.012D	Anterior displaced fracture of sternal end of left clavicle, subsequent encounter for fracture with routine healing
		S42.012G	Anterior displaced fracture of sternal end of left clavicle, subsequent encounter for fracture with delayed healing
		S42.012K	Anterior displaced fracture of sternal end of left clavicle, subsequent encounter for fracture with nonunion
		S42.012P	Anterior displaced fracture of sternal end of left clavicle, subsequent encounter for fracture with malunion
		S42.012S	Anterior displaced fracture of sternal end of left clavicle, sequela
		S42.013A	Anterior displaced fracture of sternal end of unspecified clavicle, initial encounter for closed fracture
		S42.013D	Anterior displaced fracture of sternal end of unspecified clavicle, subsequent encounter for fracture with routine healing
		S42.013G	Anterior displaced fracture of sternal end of unspecified clavicle, subsequent encounter for fracture with delayed healing
		S42.013K	Anterior displaced fracture of sternal end of unspecified clavicle, subsequent encounter for fracture with nonunion
		S42.013P	Anterior displaced fracture of sternal end of unspecified clavicle, subsequent encounter for fracture with malunion
		S42.013S	Anterior displaced fracture of sternal end of unspecified clavicle, sequela
		S42.014A	Posterior displaced fracture of sternal end of right clavicle, initial encounter for closed fracture
		S42.014D	Posterior displaced fracture of sternal end of right clavicle, subsequent encounter for fracture with routine healing
		S42.014G	Posterior displaced fracture of sternal end of right clavicle, subsequent encounter for fracture with delayed healing
		S42.014K	Posterior displaced fracture of sternal end of right clavicle, subsequent encounter for fracture with nonunion
		S42.014P	Posterior displaced fracture of sternal end of right clavicle, subsequent encounter for fracture with malunion
		S42.014S	Posterior displaced fracture of sternal end of right clavicle, sequela
		S42.015A	Posterior displaced fracture of sternal end of left clavicle, initial encounter for closed fracture
		S42.015D	Posterior displaced fracture of sternal end of left clavicle, subsequent encounter for fracture with routine healing
		S42.015G	Posterior displaced fracture of sternal end of left clavicle, subsequent encounter for fracture with delayed healing
		S42.015K	Posterior displaced fracture of sternal end of left clavicle, subsequent encounter for fracture with nonunion
		S42.015P	Posterior displaced fracture of sternal end of left clavicle, subsequent encounter for fracture with malunion
		S42.015S	Posterior displaced fracture of sternal end of left clavicle, sequela
		S42.016A	Posterior displaced fracture of sternal end of unspecified clavicle, initial encounter for closed fracture
		S42.016D	Posterior displaced fracture of sternal end of unspecified clavicle, subsequent encounter for fracture with routine healing
		S42.016G	Posterior displaced fracture of sternal end of unspecified clavicle, subsequent encounter for fracture with delayed healing
Clavicle Fracture cont.	ICD-10 cont.	S42.016K	Posterior displaced fracture of sternal end of unspecified clavicle, subsequent encounter for fracture with nonunion
		S42.016P	Posterior displaced fracture of sternal end of unspecified clavicle, subsequent encounter for fracture with malunion
		S42.016S	Posterior displaced fracture of sternal end of unspecified clavicle, sequela
		S42.017A	Nondisplaced fracture of sternal end of right clavicle, initial encounter for closed fracture
		S42.017D	Nondisplaced fracture of sternal end of right clavicle, subsequent encounter for fracture with routine healing
		S42.017G	Nondisplaced fracture of sternal end of right clavicle, subsequent encounter for fracture with delayed healing
		S42.017K	Nondisplaced fracture of sternal end of right clavicle, subsequent encounter for fracture with nonunion
		S42.017P	Nondisplaced fracture of sternal end of right clavicle, subsequent encounter for fracture with malunion
		S42.017S	Nondisplaced fracture of sternal end of right clavicle, sequela
		S42.018A	Nondisplaced fracture of sternal end of left clavicle, initial encounter for closed fracture
		S42.018D	Nondisplaced fracture of sternal end of left clavicle, subsequent encounter for fracture with routine healing
		S42.018G	Nondisplaced fracture of sternal end of left clavicle, subsequent encounter for fracture with delayed healing
		S42.018K	Nondisplaced fracture of sternal end of left clavicle, subsequent encounter for fracture with nonunion
		S42.018P	Nondisplaced fracture of sternal end of left clavicle, subsequent encounter for fracture with malunion
		S42.018S	Nondisplaced fracture of sternal end of left clavicle, sequela
		S42.019A	Nondisplaced fracture of sternal end of unspecified clavicle, initial encounter for closed fracture
		S42.019D	Nondisplaced fracture of sternal end of unspecified clavicle, subsequent encounter for fracture with routine healing
		S42.019G	Nondisplaced fracture of sternal end of unspecified clavicle, subsequent encounter for fracture with delayed healing
		S42.019K	Nondisplaced fracture of sternal end of unspecified clavicle, subsequent encounter for fracture with nonunion
		S42.019P	Nondisplaced fracture of sternal end of unspecified clavicle, subsequent encounter for fracture with malunion
		S42.019S	Nondisplaced fracture of sternal end of unspecified clavicle, sequela
		S42.021A	Displaced fracture of shaft of right clavicle, initial encounter for closed fracture
		S42.021D	Displaced fracture of shaft of right clavicle, subsequent encounter for fracture with routine healing
		S42.021G	Displaced fracture of shaft of right clavicle, subsequent encounter for fracture with delayed healing
		S42.021K	Displaced fracture of shaft of right clavicle, subsequent encounter for fracture with nonunion
		S42.021P	Displaced fracture of shaft of right clavicle, subsequent encounter for fracture with malunion
		S42.021S	Displaced fracture of shaft of right clavicle, sequela
		S42.022A	Displaced fracture of shaft of left clavicle, initial encounter for closed fracture
		S42.022D	Displaced fracture of shaft of left clavicle, subsequent encounter for fracture with routine healing
		S42.022G	Displaced fracture of shaft of left clavicle, subsequent encounter for fracture with delayed healing
		S42.022K	Displaced fracture of shaft of left clavicle, subsequent encounter for fracture with nonunion
		S42.022P	Displaced fracture of shaft of left clavicle, subsequent encounter for fracture with malunion
		S42.022S	Displaced fracture of shaft of left clavicle, sequela
		S42.023A	Displaced fracture of shaft of unspecified clavicle, initial encounter for closed fracture
Clavicle Fracture cont.	ICD-10 cont.	S42.023D	Displaced fracture of shaft of unspecified clavicle, subsequent encounter for fracture with routine healing
		S42.023G	Displaced fracture of shaft of unspecified clavicle, subsequent encounter for fracture with delayed healing
		S42.023K	Displaced fracture of shaft of unspecified clavicle, subsequent encounter for fracture with nonunion
		S42.023P	Displaced fracture of shaft of unspecified clavicle, subsequent encounter for fracture with malunion
		S42.023S	Displaced fracture of shaft of unspecified clavicle, sequela
		S42.024A	Nondisplaced fracture of shaft of right clavicle, initial encounter for closed fracture
		S42.024D	Nondisplaced fracture of shaft of right clavicle, subsequent encounter for fracture with routine healing
		S42.024G	Nondisplaced fracture of shaft of right clavicle, subsequent encounter for fracture with delayed healing
		S42.024K	Nondisplaced fracture of shaft of right clavicle, subsequent encounter for fracture with nonunion
		S42.024P	Nondisplaced fracture of shaft of right clavicle, subsequent encounter for fracture with malunion
		S42.024S	Nondisplaced fracture of shaft of right clavicle, sequela
		S42.025A	Nondisplaced fracture of shaft of left clavicle, initial encounter for closed fracture
		S42.025D	Nondisplaced fracture of shaft of left clavicle, subsequent encounter for fracture with routine healing
		S42.025G	Nondisplaced fracture of shaft of left clavicle, subsequent encounter for fracture with delayed healing
		S42.025K	Nondisplaced fracture of shaft of left clavicle, subsequent encounter for fracture with nonunion
		S42.025P	Nondisplaced fracture of shaft of left clavicle, subsequent encounter for fracture with malunion
		S42.025S	Nondisplaced fracture of shaft of left clavicle, sequela
		S42.026A	Nondisplaced fracture of shaft of unspecified clavicle, initial encounter for closed fracture
		S42.026D	Nondisplaced fracture of shaft of unspecified clavicle, subsequent encounter for fracture with routine healing
		S42.026G	Nondisplaced fracture of shaft of unspecified clavicle, subsequent encounter for fracture with delayed healing
		S42.026K	Nondisplaced fracture of shaft of unspecified clavicle, subsequent encounter for fracture with nonunion
		S42.026P	Nondisplaced fracture of shaft of unspecified clavicle, subsequent encounter for fracture with malunion
		S42.026S	Nondisplaced fracture of shaft of unspecified clavicle, sequela
		S42.031A	Displaced fracture of lateral end of right clavicle, initial encounter for closed fracture
		S42.031D	Displaced fracture of lateral end of right clavicle, subsequent encounter for fracture with routine healing
		S42.031G	Displaced fracture of lateral end of right clavicle, subsequent encounter for fracture with delayed healing
		S42.031K	Displaced fracture of lateral end of right clavicle, subsequent encounter for fracture with nonunion
		S42.031P	Displaced fracture of lateral end of right clavicle, subsequent encounter for fracture with malunion
		S42.031S	Displaced fracture of lateral end of right clavicle, sequela
		S42.032A	Displaced fracture of lateral end of left clavicle, initial encounter for closed fracture
		S42.032D	Displaced fracture of lateral end of left clavicle, subsequent encounter for fracture with routine healing
		S42.032G	Displaced fracture of lateral end of left clavicle, subsequent encounter for fracture with delayed healing
		S42.032K	Displaced fracture of lateral end of left clavicle, subsequent encounter for fracture with nonunion
		S42.032P	Displaced fracture of lateral end of left clavicle, subsequent encounter for fracture with malunion
Clavicle Fracture cont.	ICD-10 cont.	S42.032S	Displaced fracture of lateral end of left clavicle, sequela
		S42.033A	Displaced fracture of lateral end of unspecified clavicle, initial encounter for closed fracture
		S42.033D	Displaced fracture of lateral end of unspecified clavicle, subsequent encounter for fracture with routine healing
		S42.033G	Displaced fracture of lateral end of unspecified clavicle, subsequent encounter for fracture with delayed healing
		S42.033K	Displaced fracture of lateral end of unspecified clavicle, subsequent encounter for fracture with nonunion
		S42.033P	Displaced fracture of lateral end of unspecified clavicle, subsequent encounter for fracture with malunion
		S42.033S	Displaced fracture of lateral end of unspecified clavicle, sequela
		S42.034A	Nondisplaced fracture of lateral end of right clavicle, initial encounter for closed fracture
		S42.034D	Nondisplaced fracture of lateral end of right clavicle, subsequent encounter for fracture with routine healing
		S42.034G	Nondisplaced fracture of lateral end of right clavicle, subsequent encounter for fracture with delayed healing
		S42.034K	Nondisplaced fracture of lateral end of right clavicle, subsequent encounter for fracture with nonunion
		S42.034P	Nondisplaced fracture of lateral end of right clavicle, subsequent encounter for fracture with malunion
		S42.034S	Nondisplaced fracture of lateral end of right clavicle, sequela
		S42.035A	Nondisplaced fracture of lateral end of left clavicle, initial encounter for closed fracture
		S42.035D	Nondisplaced fracture of lateral end of left clavicle, subsequent encounter for fracture with routine healing
		S42.035G	Nondisplaced fracture of lateral end of left clavicle, subsequent encounter for fracture with delayed healing
		S42.035K	Nondisplaced fracture of lateral end of left clavicle, subsequent encounter for fracture with nonunion
		S42.035P	Nondisplaced fracture of lateral end of left clavicle, subsequent encounter for fracture with malunion
		S42.035S	Nondisplaced fracture of lateral end of left clavicle, sequela
		S42.036A	Nondisplaced fracture of lateral end of unspecified clavicle, initial encounter for closed fracture
		S42.036D	Nondisplaced fracture of lateral end of unspecified clavicle, subsequent encounter for fracture with routine healing
		S42.036G	Nondisplaced fracture of lateral end of unspecified clavicle, subsequent encounter for fracture with delayed healing
		S42.036K	Nondisplaced fracture of lateral end of unspecified clavicle, subsequent encounter for fracture with nonunion
		S42.036P	Nondisplaced fracture of lateral end of unspecified clavicle, subsequent encounter for fracture with malunion
		S42.036S	Nondisplaced fracture of lateral end of unspecified clavicle, sequela
Coracoid Fracture	ICD-9	811.00	Closed fracture of scapula, unspecified part
		811.02	Closed fracture of coracoid process of scapula
	ICD-10	S42.101A	Fracture of unspecified part of scapula, right shoulder, initial encounter for closed fracture
		S42.101D	Fracture of unspecified part of scapula, right shoulder, subsequent encounter for fracture with routine healing
		S42.101G	Fracture of unspecified part of scapula, right shoulder, subsequent encounter for fracture with delayed healing
		S42.101K	Fracture of unspecified part of scapula, right shoulder, subsequent encounter for fracture with nonunion
		S42.101P	Fracture of unspecified part of scapula, right shoulder, subsequent encounter for fracture with malunion
		S42.101S	Fracture of unspecified part of scapula, right shoulder, sequela
		S42.102A	Fracture of unspecified part of scapula, left shoulder, initial encounter for closed fracture
Coracoid Fracture cont.	ICD-10 cont.	S42.102D	Fracture of unspecified part of scapula, left shoulder, subsequent encounter for fracture with routine healing
		S42.102G	Fracture of unspecified part of scapula, left shoulder, subsequent encounter for fracture with delayed healing
		S42.102K	Fracture of unspecified part of scapula, left shoulder, subsequent encounter for fracture with nonunion
		S42.102P	Fracture of unspecified part of scapula, left shoulder, subsequent encounter for fracture with malunion
		S42.102S	Fracture of unspecified part of scapula, left shoulder, sequela
		S42.109A	Fracture of unspecified part of scapula, unspecified shoulder, initial encounter for closed fracture
		S42.109D	Fracture of unspecified part of scapula, unspecified shoulder, subsequent encounter for fracture with routine healing
		S42.109G	Fracture of unspecified part of scapula, unspecified shoulder, subsequent encounter for fracture with delayed healing
		S42.109K	Fracture of unspecified part of scapula, unspecified shoulder, subsequent encounter for fracture with nonunion
		S42.109P	Fracture of unspecified part of scapula, unspecified shoulder, subsequent encounter for fracture with malunion
		S42.109S	Fracture of unspecified part of scapula, unspecified shoulder, sequela
		S42.111A	Displaced fracture of body of scapula, right shoulder, initial encounter for closed fracture
		S42.111D	Displaced fracture of body of scapula, right shoulder, subsequent encounter for fracture with routine healing
		S42.111G	Displaced fracture of body of scapula, right shoulder, subsequent encounter for fracture with delayed healing
		S42.111K	Displaced fracture of body of scapula, right shoulder, subsequent encounter for fracture with nonunion
		S42.111P	Displaced fracture of body of scapula, right shoulder, subsequent encounter for fracture with malunion
		S42.111S	Displaced fracture of body of scapula, right shoulder, sequela
		S42.112A	Displaced fracture of body of scapula, left shoulder, initial encounter for closed fracture
		S42.112D	Displaced fracture of body of scapula, left shoulder, subsequent encounter for fracture with routine healing
		S42.112G	Displaced fracture of body of scapula, left shoulder, subsequent encounter for fracture with delayed healing
		S42.112K	Displaced fracture of body of scapula, left shoulder, subsequent encounter for fracture with nonunion
		S42.112P	Displaced fracture of body of scapula, left shoulder, subsequent encounter for fracture with malunion
		S42.112S	Displaced fracture of body of scapula, left shoulder, sequela
		S42.113A	Displaced fracture of body of scapula, unspecified shoulder, initial encounter for closed fracture
		S42.113D	Displaced fracture of body of scapula, unspecified shoulder, subsequent encounter for fracture with routine healing
		S42.113G	Displaced fracture of body of scapula, unspecified shoulder, subsequent encounter for fracture with delayed healing
		S42.113K	Displaced fracture of body of scapula, unspecified shoulder, subsequent encounter for fracture with nonunion
		S42.113P	Displaced fracture of body of scapula, unspecified shoulder, subsequent encounter for fracture with malunion
		S42.113S	Displaced fracture of body of scapula, unspecified shoulder, sequela
		S42.114A	Nondisplaced fracture of body of scapula, right shoulder, initial encounter for closed fracture
		S42.114D	Nondisplaced fracture of body of scapula, right shoulder, subsequent encounter for fracture with routine healing
		S42.114G	Nondisplaced fracture of body of scapula, right shoulder, subsequent encounter for fracture with delayed healing
		S42.114K	Nondisplaced fracture of body of scapula, right shoulder, subsequent encounter for fracture with nonunion
		S42.114P	Nondisplaced fracture of body of scapula, right shoulder, subsequent encounter for fracture with malunion
Coracoid Fracture cont.	ICD-10 cont.	S42.114S	Nondisplaced fracture of body of scapula, right shoulder, sequela
		S42.115A	Nondisplaced fracture of body of scapula, left shoulder, initial encounter for closed fracture
		S42.115D	Nondisplaced fracture of body of scapula, left shoulder, subsequent encounter for fracture with routine healing
		S42.115G	Nondisplaced fracture of body of scapula, left shoulder, subsequent encounter for fracture with delayed healing
		S42.115K	Nondisplaced fracture of body of scapula, left shoulder, subsequent encounter for fracture with nonunion
		S42.115P	Nondisplaced fracture of body of scapula, left shoulder, subsequent encounter for fracture with malunion
		S42.115S	Nondisplaced fracture of body of scapula, left shoulder, sequela
		S42.116A	Nondisplaced fracture of body of scapula, unspecified shoulder, initial encounter for closed fracture
		S42.116D	Nondisplaced fracture of body of scapula, unspecified shoulder, subsequent encounter for fracture with routine healing
		S42.116G	Nondisplaced fracture of body of scapula, unspecified shoulder, subsequent encounter for fracture with delayed healing
		S42.116K	Nondisplaced fracture of body of scapula, unspecified shoulder, subsequent encounter for fracture with nonunion
		S42.116P	Nondisplaced fracture of body of scapula, unspecified shoulder, subsequent encounter for fracture with malunion
		S42.116S	Nondisplaced fracture of body of scapula, unspecified shoulder, sequela
		S42.121A	Displaced fracture of acromial process, right shoulder, initial encounter for closed fracture
		S42.121D	Displaced fracture of acromial process, right shoulder, subsequent encounter for fracture with routine healing
		S42.121G	Displaced fracture of acromial process, right shoulder, subsequent encounter for fracture with delayed healing
		S42.121K	Displaced fracture of acromial process, right shoulder, subsequent encounter for fracture with nonunion
		S42.121P	Displaced fracture of acromial process, right shoulder, subsequent encounter for fracture with malunion
		S42.121S	Displaced fracture of acromial process, right shoulder, sequela
		S42.122A	Displaced fracture of acromial process, left shoulder, initial encounter for closed fracture
		S42.122D	Displaced fracture of acromial process, left shoulder, subsequent encounter for fracture with routine healing
		S42.122G	Displaced fracture of acromial process, left shoulder, subsequent encounter for fracture with delayed healing
		S42.122K	Displaced fracture of acromial process, left shoulder, subsequent encounter for fracture with nonunion
		S42.122P	Displaced fracture of acromial process, left shoulder, subsequent encounter for fracture with malunion
		S42.122S	Displaced fracture of acromial process, left shoulder, sequela
		S42.123A	Displaced fracture of acromial process, unspecified shoulder, initial encounter for closed fracture
		S42.123D	Displaced fracture of acromial process, unspecified shoulder, subsequent encounter for fracture with routine healing
		S42.123G	Displaced fracture of acromial process, unspecified shoulder, subsequent encounter for fracture with delayed healing
		S42.123K	Displaced fracture of acromial process, unspecified shoulder, subsequent encounter for fracture with nonunion
		S42.123P	Displaced fracture of acromial process, unspecified shoulder, subsequent encounter for fracture with malunion
		S42.123S	Displaced fracture of acromial process, unspecified shoulder, sequela
		S42.124A	Nondisplaced fracture of acromial process, right shoulder, initial encounter for closed fracture
		S42.124D	Nondisplaced fracture of acromial process, right shoulder, subsequent encounter for fracture with routine healing
		S42.124G	Nondisplaced fracture of acromial process, right shoulder, subsequent encounter for fracture with delayed healing
Coracoid Fracture cont.	ICD-10 cont.	S42.124K	Nondisplaced fracture of acromial process, right shoulder, subsequent encounter for fracture with nonunion
		S42.124P	Nondisplaced fracture of acromial process, right shoulder, subsequent encounter for fracture with malunion
		S42.124S	Nondisplaced fracture of acromial process, right shoulder, sequela
		S42.125A	Nondisplaced fracture of acromial process, left shoulder, initial encounter for closed fracture
		S42.125D	Nondisplaced fracture of acromial process, left shoulder, subsequent encounter for fracture with routine healing
		S42.125G	Nondisplaced fracture of acromial process, left shoulder, subsequent encounter for fracture with delayed healing
		S42.125K	Nondisplaced fracture of acromial process, left shoulder, subsequent encounter for fracture with nonunion
		S42.125P	Nondisplaced fracture of acromial process, left shoulder, subsequent encounter for fracture with malunion
		S42.125S	Nondisplaced fracture of acromial process, left shoulder, sequela
		S42.126A	Nondisplaced fracture of acromial process, unspecified shoulder, initial encounter for closed fracture
		S42.126D	Nondisplaced fracture of acromial process, unspecified shoulder, subsequent encounter for fracture with routine healing
		S42.126G	Nondisplaced fracture of acromial process, unspecified shoulder, subsequent encounter for fracture with delayed healing
		S42.126K	Nondisplaced fracture of acromial process, unspecified shoulder, subsequent encounter for fracture with nonunion
		S42.126P	Nondisplaced fracture of acromial process, unspecified shoulder, subsequent encounter for fracture with malunion
		S42.126S	Nondisplaced fracture of acromial process, unspecified shoulder, sequela
		S42.131A	Displaced fracture of coracoid process, right shoulder, initial encounter for closed fracture
		S42.131D	Displaced fracture of coracoid process, right shoulder, subsequent encounter for fracture with routine healing
		S42.131G	Displaced fracture of coracoid process, right shoulder, subsequent encounter for fracture with delayed healing
		S42.131K	Displaced fracture of coracoid process, right shoulder, subsequent encounter for fracture with nonunion
		S42.131P	Displaced fracture of coracoid process, right shoulder, subsequent encounter for fracture with malunion
		S42.131S	Displaced fracture of coracoid process, right shoulder, sequela
		S42.132A	Displaced fracture of coracoid process, left shoulder, initial encounter for closed fracture
		S42.132D	Displaced fracture of coracoid process, left shoulder, subsequent encounter for fracture with routine healing
		S42.132G	Displaced fracture of coracoid process, left shoulder, subsequent encounter for fracture with delayed healing
		S42.132K	Displaced fracture of coracoid process, left shoulder, subsequent encounter for fracture with nonunion
		S42.132P	Displaced fracture of coracoid process, left shoulder, subsequent encounter for fracture with malunion
		S42.132S	Displaced fracture of coracoid process, left shoulder, sequela
		S42.133A	Displaced fracture of coracoid process, unspecified shoulder, initial encounter for closed fracture
		S42.133D	Displaced fracture of coracoid process, unspecified shoulder, subsequent encounter for fracture with routine healing
		S42.133G	Displaced fracture of coracoid process, unspecified shoulder, subsequent encounter for fracture with delayed healing
		S42.133K	Displaced fracture of coracoid process, unspecified shoulder, subsequent encounter for fracture with nonunion
		S42.133P	Displaced fracture of coracoid process, unspecified shoulder, subsequent encounter for fracture with malunion
		S42.133S	Displaced fracture of coracoid process, unspecified shoulder, sequela
		S42.134A	Nondisplaced fracture of coracoid process, right shoulder, initial encounter for closed fracture
Coracoid Fracture cont.	ICD-10 cont.	S42.134D	Nondisplaced fracture of coracoid process, right shoulder, subsequent encounter for fracture with routine healing
		S42.134G	Nondisplaced fracture of coracoid process, right shoulder, subsequent encounter for fracture with delayed healing
		S42.134K	Nondisplaced fracture of coracoid process, right shoulder, subsequent encounter for fracture with nonunion
		S42.134P	Nondisplaced fracture of coracoid process, right shoulder, subsequent encounter for fracture with malunion
		S42.134S	Nondisplaced fracture of coracoid process, right shoulder, sequela
		S42.135A	Nondisplaced fracture of coracoid process, left shoulder, initial encounter for closed fracture
		S42.135D	Nondisplaced fracture of coracoid process, left shoulder, subsequent encounter for fracture with routine healing
		S42.135G	Nondisplaced fracture of coracoid process, left shoulder, subsequent encounter for fracture with delayed healing
		S42.135K	Nondisplaced fracture of coracoid process, left shoulder, subsequent encounter for fracture with nonunion
		S42.135P	Nondisplaced fracture of coracoid process, left shoulder, subsequent encounter for fracture with malunion
		S42.135S	Nondisplaced fracture of coracoid process, left shoulder, sequela
		S42.136A	Nondisplaced fracture of coracoid process, unspecified shoulder, initial encounter for closed fracture
		S42.136D	Nondisplaced fracture of coracoid process, unspecified shoulder, subsequent encounter for fracture with routine healing
		S42.136G	Nondisplaced fracture of coracoid process, unspecified shoulder, subsequent encounter for fracture with delayed healing
		S42.136K	Nondisplaced fracture of coracoid process, unspecified shoulder, subsequent encounter for fracture with nonunion
		S42.136P	Nondisplaced fracture of coracoid process, unspecified shoulder, subsequent encounter for fracture with malunion
		S42.136S	Nondisplaced fracture of coracoid process, unspecified shoulder, sequela
		S42.141A	Displaced fracture of glenoid cavity of scapula, right shoulder, initial encounter for closed fracture
		S42.141D	Displaced fracture of glenoid cavity of scapula, right shoulder, subsequent encounter for fracture with routine healing
		S42.141G	Displaced fracture of glenoid cavity of scapula, right shoulder, subsequent encounter for fracture with delayed healing
		S42.141K	Displaced fracture of glenoid cavity of scapula, right shoulder, subsequent encounter for fracture with nonunion
		S42.141P	Displaced fracture of glenoid cavity of scapula, right shoulder, subsequent encounter for fracture with malunion
		S42.141S	Displaced fracture of glenoid cavity of scapula, right shoulder, sequela
		S42.142A	Displaced fracture of glenoid cavity of scapula, left shoulder, initial encounter for closed fracture
		S42.142D	Displaced fracture of glenoid cavity of scapula, left shoulder, subsequent encounter for fracture with routine healing
		S42.142G	Displaced fracture of glenoid cavity of scapula, left shoulder, subsequent encounter for fracture with delayed healing
		S42.142K	Displaced fracture of glenoid cavity of scapula, left shoulder, subsequent encounter for fracture with nonunion
		S42.142P	Displaced fracture of glenoid cavity of scapula, left shoulder, subsequent encounter for fracture with malunion
		S42.142S	Displaced fracture of glenoid cavity of scapula, left shoulder, sequela
		S42.143A	Displaced fracture of glenoid cavity of scapula, unspecified shoulder, initial encounter for closed fracture
		S42.143D	Displaced fracture of glenoid cavity of scapula, unspecified shoulder, subsequent encounter for fracture with routine healing
		S42.143G	Displaced fracture of glenoid cavity of scapula, unspecified shoulder, subsequent encounter for fracture with delayed healing
		S42.143K	Displaced fracture of glenoid cavity of scapula, unspecified shoulder, subsequent encounter for fracture with nonunion
		S42.143P	Displaced fracture of glenoid cavity of scapula, unspecified shoulder, subsequent encounter for fracture with malunion
Coracoid Fracture cont.	ICD-10 cont.	S42.143S	Displaced fracture of glenoid cavity of scapula, unspecified shoulder, sequela
		S42.144A	Nondisplaced fracture of glenoid cavity of scapula, right shoulder, initial encounter for closed fracture
		S42.144D	Nondisplaced fracture of glenoid cavity of scapula, right shoulder, subsequent encounter for fracture with routine healing
		S42.144G	Nondisplaced fracture of glenoid cavity of scapula, right shoulder, subsequent encounter for fracture with delayed healing
		S42.144K	Nondisplaced fracture of glenoid cavity of scapula, right shoulder, subsequent encounter for fracture with nonunion
		S42.144P	Nondisplaced fracture of glenoid cavity of scapula, right shoulder, subsequent encounter for fracture with malunion
		S42.144S	Nondisplaced fracture of glenoid cavity of scapula, right shoulder, sequela
		S42.145A	Nondisplaced fracture of glenoid cavity of scapula, left shoulder, initial encounter for closed fracture
		S42.145D	Nondisplaced fracture of glenoid cavity of scapula, left shoulder, subsequent encounter for fracture with routine healing
		S42.145G	Nondisplaced fracture of glenoid cavity of scapula, left shoulder, subsequent encounter for fracture with delayed healing
		S42.145K	Nondisplaced fracture of glenoid cavity of scapula, left shoulder, subsequent encounter for fracture with nonunion
		S42.145P	Nondisplaced fracture of glenoid cavity of scapula, left shoulder, subsequent encounter for fracture with malunion
		S42.145S	Nondisplaced fracture of glenoid cavity of scapula, left shoulder, sequela
		S42.146A	Nondisplaced fracture of glenoid cavity of scapula, unspecified shoulder, initial encounter for closed fracture
		S42.146D	Nondisplaced fracture of glenoid cavity of scapula, unspecified shoulder, subsequent encounter for fracture with routine healing
		S42.146G	Nondisplaced fracture of glenoid cavity of scapula, unspecified shoulder, subsequent encounter for fracture with delayed healing
		S42.146K	Nondisplaced fracture of glenoid cavity of scapula, unspecified shoulder, subsequent encounter for fracture with nonunion
		S42.146P	Nondisplaced fracture of glenoid cavity of scapula, unspecified shoulder, subsequent encounter for fracture with malunion
		S42.146S	Nondisplaced fracture of glenoid cavity of scapula, unspecified shoulder, sequela
		S42.151A	Displaced fracture of neck of scapula, right shoulder, initial encounter for closed fracture
		S42.151D	Displaced fracture of neck of scapula, right shoulder, subsequent encounter for fracture with routine healing
		S42.151G	Displaced fracture of neck of scapula, right shoulder, subsequent encounter for fracture with delayed healing
		S42.151K	Displaced fracture of neck of scapula, right shoulder, subsequent encounter for fracture with nonunion
		S42.151P	Displaced fracture of neck of scapula, right shoulder, subsequent encounter for fracture with malunion
		S42.151S	Displaced fracture of neck of scapula, right shoulder, sequela
		S42.152A	Displaced fracture of neck of scapula, left shoulder, initial encounter for closed fracture
		S42.152D	Displaced fracture of neck of scapula, left shoulder, subsequent encounter for fracture with routine healing
		S42.152G	Displaced fracture of neck of scapula, left shoulder, subsequent encounter for fracture with delayed healing
		S42.152K	Displaced fracture of neck of scapula, left shoulder, subsequent encounter for fracture with nonunion
		S42.152P	Displaced fracture of neck of scapula, left shoulder, subsequent encounter for fracture with malunion
		S42.152S	Displaced fracture of neck of scapula, left shoulder, sequela
		S42.153A	Displaced fracture of neck of scapula, unspecified shoulder, initial encounter for closed fracture
		S42.153D	Displaced fracture of neck of scapula, unspecified shoulder, subsequent encounter for fracture with routine healing
		S42.153G	Displaced fracture of neck of scapula, unspecified shoulder, subsequent encounter for fracture with delayed healing
Coracoid Fracture cont.	ICD-10 cont.	S42.153K	Displaced fracture of neck of scapula, unspecified shoulder, subsequent encounter for fracture with nonunion
		S42.153P	Displaced fracture of neck of scapula, unspecified shoulder, subsequent encounter for fracture with malunion
		S42.153S	Displaced fracture of neck of scapula, unspecified shoulder, sequela
		S42.154A	Nondisplaced fracture of neck of scapula, right shoulder, initial encounter for closed fracture
		S42.154D	Nondisplaced fracture of neck of scapula, right shoulder, subsequent encounter for fracture with routine healing
		S42.154G	Nondisplaced fracture of neck of scapula, right shoulder, subsequent encounter for fracture with delayed healing
		S42.154K	Nondisplaced fracture of neck of scapula, right shoulder, subsequent encounter for fracture with nonunion
		S42.154P	Nondisplaced fracture of neck of scapula, right shoulder, subsequent encounter for fracture with malunion
		S42.154S	Nondisplaced fracture of neck of scapula, right shoulder, sequela
		S42.155A	Nondisplaced fracture of neck of scapula, left shoulder, initial encounter for closed fracture
		S42.155D	Nondisplaced fracture of neck of scapula, left shoulder, subsequent encounter for fracture with routine healing
		S42.155G	Nondisplaced fracture of neck of scapula, left shoulder, subsequent encounter for fracture with delayed healing
		S42.155K	Nondisplaced fracture of neck of scapula, left shoulder, subsequent encounter for fracture with nonunion
		S42.155P	Nondisplaced fracture of neck of scapula, left shoulder, subsequent encounter for fracture with malunion
		S42.155S	Nondisplaced fracture of neck of scapula, left shoulder, sequela
		S42.156A	Nondisplaced fracture of neck of scapula, unspecified shoulder, initial encounter for closed fracture
		S42.156D	Nondisplaced fracture of neck of scapula, unspecified shoulder, subsequent encounter for fracture with routine healing
		S42.156G	Nondisplaced fracture of neck of scapula, unspecified shoulder, subsequent encounter for fracture with delayed healing
		S42.156K	Nondisplaced fracture of neck of scapula, unspecified shoulder, subsequent encounter for fracture with nonunion
		S42.156P	Nondisplaced fracture of neck of scapula, unspecified shoulder, subsequent encounter for fracture with malunion
		S42.156S	Nondisplaced fracture of neck of scapula, unspecified shoulder, sequela
		S42.191A	Fracture of other part of scapula, right shoulder, initial encounter for closed fracture
		S42.191D	Fracture of other part of scapula, right shoulder, subsequent encounter for fracture with routine healing
		S42.191G	Fracture of other part of scapula, right shoulder, subsequent encounter for fracture with delayed healing
		S42.191K	Fracture of other part of scapula, right shoulder, subsequent encounter for fracture with nonunion
		S42.191P	Fracture of other part of scapula, right shoulder, subsequent encounter for fracture with malunion
		S42.191S	Fracture of other part of scapula, right shoulder, sequela
		S42.192A	Fracture of other part of scapula, left shoulder, initial encounter for closed fracture
		S42.192D	Fracture of other part of scapula, left shoulder, subsequent encounter for fracture with routine healing
		S42.192G	Fracture of other part of scapula, left shoulder, subsequent encounter for fracture with delayed healing
		S42.192K	Fracture of other part of scapula, left shoulder, subsequent encounter for fracture with nonunion
		S42.192P	Fracture of other part of scapula, left shoulder, subsequent encounter for fracture with malunion
		S42.192S	Fracture of other part of scapula, left shoulder, sequela
		S42.199A	Fracture of other part of scapula, unspecified shoulder, initial encounter for closed fracture
Coracoid Fracture cont.	ICD-10 cont.	S42.199D	Fracture of other part of scapula, unspecified shoulder, subsequent encounter for fracture with routine healing
		S42.199G	Fracture of other part of scapula, unspecified shoulder, subsequent encounter for fracture with delayed healing
		S42.199K	Fracture of other part of scapula, unspecified shoulder, subsequent encounter for fracture with nonunion
		S42.199P	Fracture of other part of scapula, unspecified shoulder, subsequent encounter for fracture with malunion
		S42.199S	Fracture of other part of scapula, unspecified shoulder, sequela
